# HIV, human rights and the last mile

**DOI:** 10.1002/jia2.25434

**Published:** 2019-12-09

**Authors:** Joseph J Amon, Nina Sun

**Affiliations:** ^1^ Office of Global Health Dornsife School of Public Health Drexel University Philadelphia PA USA

**Keywords:** human rights, HIV, key populations, drugs, prisoners, disability, women

In 1996, OHCHR and UNAIDS adopted the International Guidelines on HIV/AIDS and Human Rights 1. They were intended to provide a framework for a rights‐based response to the HIV epidemic and recognized that an extensive array of human rights are critical to a successful HIV response (Box 1). Twenty‐three years later, as is often the case in the fight for human rights, much has changed, but much still needs to change to ensure respect for human rights, to uphold the principles outlined in the Guidelines, and to bring to scale rights‐based HIV programmes.

Box 1 **Key Human Rights Relevant to the HIV Response 1**

The rights to non‐discrimination, equal protection and equality before the law;

The right to life;

The right to the highest attainable standard of physical and mental health;

The right to liberty and security of person;

The right to freedom of movement;

The right to seek and enjoy asylum;

The right to privacy;

The right to freedom of opinion and expression and the right to freely receive and impart information;

The right to freedom of association;

The right to work;

The right to marry and to found a family;

The right to equal access to education;

The right to an adequate standard of living;

The right to social security, assistance and welfare;

The right to share in scientific advancement and its benefits;

The right to participate in public and cultural life;

The right to be free from torture and cruel, inhuman or degrading treatment or punishment.

Human rights are now, at least rhetorically, central to the strategies of many UN and donor agencies. Many countries have also acknowledged that respecting, protecting and fulfilling human rights are important to achieving the global goals of ending the HIV epidemic and leaving no one behind. In practice, however, governments continue to criminalize key populations (such as sex workers, lesbian, gay, bisexual, transgender, queer or questioning and intersex (LGBTQI) individuals and persons who use drugs), deny them access to HIV prevention and treatment services and block research examining human rights of key populations. Despite recognition of the pernicious effects of stigma and discrimination since the start of the epidemic 2, funding for legal and policy reforms and efforts to create discrimination‐free health centres is often zero or close to it. Most donors remain uncomfortable talking about accountability in the face of human rights violations, and the States responsible are happy not to be held to account. The result is a global HIV response that falls short of its goals as well as its potential to reduce broader health disparities.

For example, it is estimated that people who inject drugs have 36 times the risk of acquiring HIV than adults in the general population 3. While there has been some progress on the political level, such as the development of the UN System Common Position on drug‐related matters 4, and the International Guidelines on Drug Policy and Human Rights 5, the most recent available data from 2014, from Cambodia, China, Lao PDR, Malaysia, the Philippines, Thailand and Viet Nam, for example, showed that over 450,000 people were arbitrarily detained, often subjected to cruel or inhuman treatment, denied access to evidence‐based drug dependency treatment and may be unable to access HIV care 6. This is despite the 2012 statement by 12 UN agencies calling for the closure of compulsory drug detention centres 7. Globally, prisoners are estimated to have an HIV prevalence five times that in the general population and have limited access to HIV prevention or treatment 3. Non‐nationals in particular often face discriminatory policies and denial of care when imprisoned, such as has been recently reported in the United Arab Emirates 8. In both drug detention centres and prisons, human rights law is clear that while detainees may suffer restrictions on their liberty, they retain their right to health and other rights. The UN Standard Minimum Rules for the Treatment of Prisoners, revised in 2015 as the Mandela Rules, reiterate these obligations 9. States and donors should recognize that as nearly all detainees are released and return to their communities, the lack of investment and attention to the human rights of this population undermines the progress achieved outside of detention settings.

Another population that the HIV response has largely ignored is persons with disabilities, who remain among the world's most stigmatized, and often face systematic violations of rights to education, housing and employment as well as health 10. They often have limited access to information on sexual education and “safe sex,” struggle to access legal and social protection, are at increased risk of violence and sexual abuse 11. Despite numerous calls to mainstream disability into the HIV response, prevention and treatment services remain inaccessible for many persons with disabilities. In addition, few studies of HIV prevalence disaggregate persons with disabilities or examine their specific determinants of vulnerability or characteristics of effective prevention programmes 12.

Twenty‐five years after the first International Conference on Population and Development in Cairo in 1994, gender inequality and the low socioeconomic status of women and girls create barriers to access to the full range of sexual and reproductive health services and contribute to HIV vulnerability. In sub‐Saharan Africa, three of the four new infections among young people aged 10 to 19 are girls 13. HIV incidence among female sex workers is 21 times higher than in the general population 13. Yet, recognition within the HIV response of the scale and the specific contribution of violence against women to HIV vulnerability is sometimes missing, even though women who have experienced physical or sexual intimate partner violence are estimated to be 50% more likely to be living with HIV 14. This is an urgent, global, reality, with one in three women reporting having experienced physical or sexual violence in their lifetime 14. Data from India, Nicaragua, Romania and Uganda, for example, have demonstrated levels of physical and sexual violence against women ranging from 23% to 50% 15.

Despite these dire statistics, there are steps being taken to expand human rights programs that can reduce HIV vulnerability. The Global Fund's 2017 to 2022 Strategy explicitly prioritizes the promotion of human rights and gender equality as essential steps to achieving its goal of ending HIV, tuberculosis and malaria, and provides dedicated funding for programmes to reduce human rights‐related barriers to health services. A report issued in November 2019 from the Global Fund's Office of the Inspector General assessed its progress and presented a series of recommendations that all donor and implementing agencies should closely examine 16. For example, it suggests developing differentiated support frameworks for each country according to its human rights environment, putting human rights technical experts in the grant making staff and improving monitoring and evaluation on human rights‐related investments. These are small steps that could make a big difference.

The success of the HIV response to date has been founded on human rights principles, such as recognizing the equal dignity and worth of all persons, as well as the meaningful involvement of affected communities and people living with HIV (GIPA). This participation, from the design and implementation of local programmes to the global governance of such institutions as UNAIDS and the Global Fund, has contributed to more effective responses and attention to gaps and challenges that would otherwise be overlooked. However, not all of those affected have been equally included, and the gaps identified in this Viewpoint reflect populations where more engagement is needed. To reach “the last mile” we must reach out to those who remain marginalized and excluded and recommit to their engagement and to recognizing their rights, equality and dignity.

## Competing interests

The authors have no competing interest.

## Authors' contributions

JJA conceptualized and drafted the commentary. NS provided substantial feedback on the draft and subsequent revisions.

## Authors' information

Joseph J. Amon is the Director of Global Health and Director of the Jonathan Mann Global Health and Human Rights Initiative at the Dornsife School of Public Health at Drexel University. He serves as co‐chair of the UNAIDS Human Rights Reference Group on HIV and Human Rights and is a member of the Working Group on Monitoring and Evaluating Programmes to Remove Human Rights Barriers to HIV, TB and Malaria Services of the Global Fund. Nina Sun is the Deputy Director of the Jonathan Mann Global Health and Human Rights Initiative at the Dornsife School of Public Health at Drexel University.
